# Outcome evaluation of COVID-19 infected patients by disease symptoms: a cross-sectional study in Ilam Province, Iran

**DOI:** 10.1186/s12879-021-06613-7

**Published:** 2021-09-03

**Authors:** Jamil Sadeghifar, Habib Jalilian, Khalil Momeni, Hamed Delam, Tadesse Sheleme, Ayoub Rashidi, Fariba Hemmati, Shahab Falahi, Morteza Arab-Zozani

**Affiliations:** 1 Department of Public Health and Health Education, School of Health, Ilam University of Medical Sciences, Ilam, Iran; 2 Social Determinants of Health Research Center (SDHRC), Department of Health Services Management, School of Health, Ahvaz Jundishapur University of Medical Sciences, Ahvaz, Iran; 3 Student Research Committee, Larestan University of Medical Sciences, Larestan, Iran; 4 Department of Pharmacy, College of Health Science, Mettu University, Metu, Ethiopia; 5 Ilam University of Medical Sciences, Ilam, Iran; 6 Emergency Department, Imam Khomeini Hospital, Ilam University of Medical Sciences, Ilam, Iran; 7 Zoonotic Diseases Research Center, Ilam University of Medical Sciences, Ilam, Iran; 8 Social Determinants of Health Research Center, Birjand University of Medical Sciences, Birjand, Iran

**Keywords:** COVID-19, SARS-COV-2, Symptoms, Outcome, Iran

## Abstract

**Background:**

Novel coronavirus disease-19 (COVID-19) was declared as a global pandemic in 2020. With the spread of the disease, a better understanding of patient outcomes associated with their symptoms in diverse geographic levels is vital. This study aimed to evaluate clinical outcomes of COVID-19 patients by disease symptoms in Ilam province, Iran.

**Methods:**

This was a cross-sectional study. Data were collected from integrated health system records for all hospitals affiliated with the Ilam University of Medical Sciences between 26-Jan-2020 and 02-May-2020. All patients with a confirmed positive test were included in this study. Descriptive analyses, chi-square test, and binary logistic regression model were performed by using SPSS version 22.

**Results:**

The mean age of participants was 46.47 ± 18.24 years. Of the 3608 patients, 3477 (96.1%) were discharged, and 129 (3.9%) died. 54.2% of the patients were male and were in the age group of 30–40 years. Cough, sore throat, shortness of breath or difficulty breathing, and fever or chills were the most common symptoms. Patients with symptoms of shortness of breath, abnormal radiographic findings of the chest, and chest pain and pressure were relatively more likely to die. According to binary logistic regression results, the probability of death in patients with shortness of breath, abnormal chest radiographic findings, and chest pain was 1.34, 1.24, and 1.32 times higher, respectively, than for those without.

**Conclusion:**

Our study provides evidence that the presentation of some symptoms significantly impacts outcomes of patients infected with SARS-CoV-2. Early detection of symptoms and proper management of outcomes can reduce mortality in patients with COVID-19.

## Background

In late December 2019, the first cases of pneumonia of unknown etiology were reported in Wuhan, China [1]. After a while, a novel Coronavirus, severe acute respiratory syndrome coronavirus 2 (SARS-COV-2), was identified as the causative agent and was subsequently named novel Coronavirus disease 2019 (COVID-19) by the World Health Organization (WHO) [2]. A few months after the onset of the disease, the WHO has declared the outbreak a global pandemic [3]. More than 210 million cases worldwide and 4 million cases in Iran have been reported so far, of which more than 4 million deaths have been recorded in the world, representing a significant statistic compared to previous pandemics [4, 5]. To date (18.08.2021), according to statistics, Iran ranks 14th in terms of the number of cases and 9^th^ globally in terms of the number of deaths [5]. Considering the specific features of this disease and its rapid expansion globally, especially in Iran, identifying the epidemiological characteristics of patients and their clinical features is highly important to health planners and policymakers to make effective decisions to control or prevent the epidemic [6]. Although the main features of COVID-19 have been previously reported [7, 8], it is essential to look at the characteristics of individuals in specific populations. Given that epidemiological characteristics of patients in a particular region can be different from other parts of the world, knowledge of these epidemiological and clinical characteristics can be helpful to local authorities to provide the necessary facilities and take measures to control the spread of the disease [9–11]. This study, therefore, aimed to evaluate the disease outcomes in COVID-19 infected patients by disease symptoms in Ilam province, west of Iran.

## Methods

### Study design, patients, and data collection

This was a cross-sectional study. Data were collected at all hospitals affiliated with the Ilam University of Medical Sciences. All suspected individuals who were referred to the hospitals with a confirmed test, and their information were registered in integrated health system records of Iran entitled “SIB” and related to Ilam province, between 26-Jan-2020 and 02-May-2020 were included in our study. The extracted information was as follows: sex, age, national code, date of admission and hospitalization, signs and symptoms, contact history, type of comorbidities, test results, and final outcome. Positive cases of COVID-19 were confirmed by SARS-CoV-2 real-time reverse transcriptase-polymerase chain reaction (RT-PCR). Data were collected from four hospitals that provided care for these patients. All information was kept confidential.

The study was approved by the Ethics Committee of the Ilam University of Medical Sciences (Reference No. IR.MEDILAM.REC.1399.043).

### Statistical analysis

Data retrieved were entered into Microsoft Excel (version 13) and analyzed using SPSS software version 22.0. Two researchers independently reviewed the extracted data for accuracy. Descriptive analyses of the variables were reported as mean (Mean ± SD) or percentage (%). A Chi-square test was used to evaluate the effect of demographic variables (age, sex, etc.) and underlying diseases/symptoms on the outcome of the infected patients. Also, the cumulative effect of all independent variables on disease outcome was investigated using binary logistic regression (Table 1).

**Table 1:** Frequency of sex and age groups

Variables	Mood	Frequency	Percent
Sex	Male	1955	54.2
	Female	1653	45.8
Age groups	< 10	55	1.9
	10–20	56	1.9
	20–30	433	14.8
	30–40	813	27.8
	40–50	645	22.0
	50–60	444	15.2
	> 60	480	16.4

## Results

The study population consisted of 3608 confirmed COVID-19 cases, with a mean age of 46.47 ± 18.24 years. Among the patients in our study, 3477 (96.1%) were discharged, and 129 (3.9%) died. Just over half (54.2%) of all case patients were male (54.2%) and 30–40 years of age. As shown in Table 2, cough, sore throat, shortness of breath or difficulty breathing, and fever or chills were the most common symptoms.

**Table 2:** Frequency of disease symptoms

Symptoms	Frequency	Percent
Cough	1465	40.6
Shortness of breath or difficulty breathing	949	26.3
Sore throat	931	25.8
Fever or chills	866	24.0
Muscle or body aches	401	11.1
Headache	339	9.4
General weakness	237	6.6
Nausea or vomiting	120	3.3
Diarrhea	115	3.2
Abnormal findings of chest radiography	98	2.7
Conjunctivitis	91	2.5
New confusion	85	2.4
Persistent pain or pressure in the chest	53	1.5
Stomach ache	52	1.4
Congestion or runny nose	22	0.6
External pharynx	18	0.5
Eye redness	9	0.2

Table 3 presents the results of Chi-square and Fisher’s Exact Test. People with symptoms of shortness of breath, abnormal chest radiograph findings, and chest pain and pressure were relatively more likely to die. Conversely, those with symptoms of sore throat, headache, diarrhea, contusions, and muscle pain were relatively less likely to die.

**Table 3:** Association between disease symptoms and disease outcome

Symptoms	Discharge	Death	X^2^	P-value
Shortness of breath or difficulty breathing	22.8%	56.6%	77.26	< 0.0001**
Abnormal findings of chest radiography	1.7%	10.1%	42.81	< 0.0001**
Persistent pain or pressure in the chest	1.4%	4.7%	8.52	0.004**
Sore throat	27.0%	11.6%	15.02	< 0.0001**
Headache*	10.1%	2.3%	8.45	0.001**
Diarrhea*	3.5%	0.0%	4.66	0.02**
Muscle or body aches	12.0%	3.9%	7.89	0.005**
Fever or chills	24.7%	17.8%	3.17	0.075
Eye redness*	0.3%	0.0%	0.36	1.00
Cough	39.3%	39.5%	0.002	0.96
General weakness	6.2%	9.3%	2.004	0.15
Nausea or vomiting*	3.5%	2.3%	0.48	0.62
Conjunctivitis*	2.7%	2.3%	0.059	1.00
New confusion*	2.6%	0.8%	1.65	0.37
*Abnormal lung sound	0.1%	0.8%	3.45	0.18
Stomach ache*	1.6%	0.0%	2.105	0.26
Congestion or runny nose*	0.6%	0.0%	0.81	1.00
External pharynx*	0.6%	0.0%	0.73	1.00

*In cases where expected counts were less than 5, Fisher’s exact test was used instead of the Chi-square test

**P < 0.05 was considered as significant

Table 4 reports the results of the binary logistic regression model. As evident in the table, shortness of breath, abnormal chest radiograph findings, and chest pressure and pain predicted the outcome of death in patients. Moreover, patients with symptoms of shortness of breath, abnormal findings on chest radiography, and chest pain and pressure were, respectively, 1.34, 1.24, and 1.32 times higher, more likely to die than those without these symptoms.

**Table 4:** Binary logistic regression of disease outcome by disease symptoms

Predictors	B	P-value	Wald	Exp (B)
Shortness of breath or difficulty breathing	1.344	< 0.0001*	49.940	3.834
Persistent pain or pressure in the chest	1.242	0.011	6.523	3.464
Abnormal findings of chest radiography	1.320	< 0.0001*	13.541	3.745
Sore throat	− 0.675	0.02*	5.452	0.509
Headache	− 1.177	0.057	3.609	0.308
Body pain	− 0.880	0.070	3.289	0.415
Fever and chill	− 0.302	0.212	1.555	0.739
Cough	− 0.192	0.334	0.931	0.825
General weakness	0.457	0.170	1.880	1.580
New confusion	− 1.056	0.305	1.051	0.348
Congestion or runny nose	− 17.239	0.998	0.000	0.000
Diarrhea	− 17.786	0.996	0.000	0.000
Nausea and vomiting	0.404	0.511	0.432	1.498
Stomach ache	− 17.092	0.997	0.000	0.000
Conjunctivitis	0.932	0.143	2.146	2.539
External pharynx	− 16.458	0.999	0.000	0.000
Redness eyes	− 1.186	1.000	0.000	0.305
Constant	− 3.435	0.000*	437.435	0.032

*P < 0.05 was considered as significant

## Discussion

This study was designed to evaluate the outcome of the disease in patients with COVID-19 based on the disease symptoms. From Jan to May 2020, 3608 confirmed cases of COVID-19 were diagnosed in Ilam province, Iran; of these, 3.9% died. A previous study showed a significant increase in COVID-19 cases and deaths worldwide [12]. Shahriarirad et al. conducted a multicenter retrospective study to evaluate the clinical features of COVID-19 patients in Fars province, southern Iran. Their results showed an overall 8% mortality rate among patients with COVID-19 [13]. Furthermore, a meta-analysis study showed a 5% mortality in patients [14], representing a higher percentage than the results of our study. The differences in the obtained results can be attributed to differences in the demographic characteristics of the patients and the severity of the disease.

In our study, the majority of infected individuals were men. Women appear to be less susceptible to viral infections because of their protection against the X chromosome and sex hormones that play a crucial role in their innate immunity [15]. We also observed that patients aged 30–40 years accounted for the highest number of infected cases. A similar study in Iran (2020) showed that patients aged 50–60 years accounted for the highest incidence of COVID-19, while the highest fatality rate of the disease was among those ≥ 80 (19.27%) and 70–80 (14.85%) years old [6]. It seems that as age increases, the probability of death due to COVID-19 will increase. A retrospective, multicenter cohort study in China demonstrated that patients who died due to COVID-19 had a significantly higher mean age (about 69 years) than the patients who were surviving [16].

The logistic regression model results in the present study showed that symptoms such as shortness of breath, pain, and chest pressure, abnormal findings of chest radiography, and sore throat were the most critical factors in predicting the outcome of death in patients with COVID-19. We also found that patients who had a higher percentage of shortness of breath, chest pain and pressure, and abnormal chest radiographic findings were more likely to die than other patients, although no significant difference was observed between the two groups of survived and of the dead. A study by Chang et al. in China showed that symptoms such as fever and chills were significant parameters predicting the progression to severe stage COVID-19 [17]. Another study in China showed that the rate of severe clinical type COVID-19 and shortness of breath were significantly higher in patients aged ≥ 60 years compared to younger patients [18]. Moreover, Zhang et al. demonstrated that 100% of patients with COVID-19 whose lungs were damaged were more likely to died [19]. Li et al. also showed that the prevalence of symptoms such as cough, sputum, chest pain, and shortness of breath in patients with severe or acute COVID-19 was significantly higher than those of ordinary patients. The authors also found the CT scores of the severe/critical patients were significantly higher (7 times higher) than those of the ordinary patients [20]. A review study by GalloMarin et al. showed that hypoxia and specific CT scan findings indicate an extensive lung involvement associated with increased disease severity or death [14]. Chest imaging results in a study showed that the rate of ICU admission was higher among older patients and those with pulmonary fibrosis [21]. On the contrary, in a study by Guan et al., 20.1% of all patients with COVID-19 who had a positive RT-PCR test and had symptoms of the disease were normal in terms of chest CT scan [7]. It is, therefore, important to note that CT scan alone cannot be a diagnostic criterion for COVID-19 [22].

Another study showed that fever and shortness of breath were significantly higher in patients who died of COVID-19 than in those who survived [23]. Another similar study found that the prevalence of symptoms such as shortness of breath, chest tightness, fatigue, muscle pain, and dizziness was higher in patients with severe or critical COVID-19. Only in terms of shortness of breath, there was a significant difference between the group that died and the group that survived. The percentage of shortness of breath was significantly higher in patients who died of COVID-19 than in others [24].

## Conclusion

The results of our study showed that symptoms such as shortness of breath, pain, and chest pressure, abnormal findings of chest radiography, and sore throat were the most predictors of the outcome of death in patients with COVID-19. Patients with a higher percentage of shortness of breath, chest pain and pressure, and abnormal chest radiographic findings were more likely to die than other patients.

Therefore, given the hospitalization rate in people with the above symptoms in the hospital, training medical staff, especially nurses and operating room staff, can increase their readiness and skills in dealing with patients with shortness of breath, chest pressure, and abnormal findings of chest radiography and throat. Hospital equipping strategies such as adding oxygen generators, ECMO devices to support patients’ lungs temporarily and damaged hearts, as well as portable digital radiology devices for the respiratory ICU, are essential to reduce in-hospital traffic and expedite diagnostic and therapeutic procedures.

## Data Availability

The datasets used and/or analyzed during the current study are available from the corresponding author on reasonable request.

## Abbreviations

SARS-COV-2: Severe acute respiratory syndrome coronavirus 2
COVID-19: Novel coronavirus disease 2019
RT-PCR: Real-time reverse transcriptase-polymerase chain reaction
WHO: World Health Organization
ICU: Intensive care unit
